# Rules for resolving Mendelian inconsistencies in nuclear pedigrees typed for two-allele markers

**DOI:** 10.1371/journal.pone.0172807

**Published:** 2017-03-02

**Authors:** Sajjad Ahmad Khan, Sadaf Manzoor, Alamgir, Amjad Ali, Dost Muhammad Khan, Umair Khalil

**Affiliations:** 1 Department of Statistics, Abdul Wali Khan University Mardan, Khyber Pukhtunkhwa, Pakistan; 2 Department of Statistics, Islamia College, Peshawar, Khyber Pukhtunkhwa, Pakistan; 3 Department of Statistics, University of Peshawar, Khyber Pukhtunkhwa, Pakistan; University of Tampere, FINLAND

## Abstract

Gene-mapping studies, regularly, rely on examination for Mendelian transmission of marker alleles in a pedigree as a way of screening for genotyping errors and mutations. For analysis of family data sets, it is, usually, necessary to resolve or remove the genotyping errors prior to consideration. At the Center of Inherited Disease Research (CIDR), to deal with their large-scale data flow, they formalized their data cleaning approach in a set of rules based on PedCheck output. We scrutinize via carefully designed simulations that how well CIDR’s data cleaning rules work in practice. We found that genotype errors in siblings are detected more often than in parents for less polymorphic SNPs and vice versa for more polymorphic SNPs. Through computer simulations, we conclude that some of the CIDR’s rules work poorly in some circumstances, and we suggest a set of modified data cleaning rules that may work better than CIDR’s rules.

## Introduction

A genotyping error arises when the observed genotype differs from the true underlying genotype [1, 2]. Even with the most modern techniques, the observed genotype does not always match the true underlying genotype, and this has been shown to occur at a rate of 0.5–7% for microsatellite markers [3]. Error rates are influenced by a number of factors, but are generally quoted between 0.25% and 1% for microsatellite genotyping [4]. Where family information is available, a proportion of genotyping errors can be detected as Mendelian inconsistencies, but this is more difficult for single nucleotide polymorphism (SNP) markers with only two alleles [5]. Several authors have shown that even a small error rate (i.e., 1–2%) can have a massive impact on linkage results [6–9].

In linkage and association analysis, investigators and researchers are fully aware of the consequences of genotyping errors at the marker loci [6, 10–13]. It is well known that misspecified marker allele frequencies, genotyping errors and Mendelian inconsistencies can lead to a systematic increase in false-positive rates. Power may be reduced, and parameter estimates may be biased and/or inconsistent [1–2, 7, 14–19].

For analysis of family data, it is, usually, necessary to resolve or remove the genotyping errors prior to analysis. There are no hard and fast rules about how to clean genotyping errors from pedigree data. Researchers, typically, clean their data using: PedCheck [20], MERLIN [21], MENDEL [22], SimWalk2 [23], or Sibmed [7].

At the Center of Inherited Disease Research (CIDR), to deal with their large-scale data flow; they formalized their data cleaning approach in a set of rules, which are presented in Table 1. They use PedCheck [20] to detect the inconsistencies, and then specific cleaning rules are triggered by certain combination of error messages from PedCheck. Here, we examine, via carefully designed simulations how well CIDR’s data cleaning rules work in practice by answering the following three questions: i) How often are genotyping errors detected?; ii) How often are these rules applied?; iii) How often are these rules applied correctly?

**Table 1:** CIDR's rules for removing Mendelian inconsistencies.

Situation in a Nuclear Family	Error Messages	Actions	Short Rule Name^a^
1 parent is inconsistent with 1 child	ERROR: Child 01 and Mother are inconsistent	Zero out the child’s genotype	1P1C:C_0_
	OR		
	ERROR: Child 01 and Father are inconsistent.		
1 parent is inconsistent with 2+ children	ERROR: Child 01 and Mother are inconsistent	Zero out the specific parent genotype	1P2+C:P_0_
	AND		
	ERROR: Child 02 and Mother are inconsistent		
	OR		
	ERROR: Child 01 and Father are inconsistent		
	AND		
	ERROR: Child 02 and Father are inconsistent.		
2 parents are inconsistent with 1 child	ERROR: Child 01 is consistent with each parent separately, but not as a pair	Zero out the child’s genotype	2P1C:C_0_
	OR		
	ERROR: Child 01 and Mother are inconsistent		
	AND		
	ERROR: Child 01 and Father are inconsistent.		
2 parents are inconsistent with 2+ children	ERROR: Child 01 is consistent with each parent separately, but not as a pair	Zero out the genotypes of the whole nuclear family	2P2+C:W_0_
	AND		
	ERROR: Child 02 is consistent with each parent separately, but not as a pair.		
	OR		
	ERROR: Child 01 and Father are inconsistent.		
	AND		
	ERROR: Child 02 and Mother are inconsistent.		

^a^Let P = parent, C = child, and W = whole family, then we name CIDR’s rules as 1. 1P1C:C_0_, 2. 1P2+C:P_0_, 3. 2P1C:C_0_, 4. 2P2+C:W_0_, where C_0_ = zero out the child’s genotype, P_0_ = zero out the specific parent genotype, and W_0_ = zero out the genotypes of the whole nuclear family.

## Methods

We wish to evaluate how well the CIDR rules (see Table 1) work in terms of accurately removing Mendelian inconsistencies from the data set. The Center of Inherited Disease Research (CIDR), routinely, uses PedCheck to detect Mendelian inconsistencies for each SNP. PedCheck [20] offers different error-checking levels: Level 0 checks for formatting errors in the pedigree structure data, Level 1 checks for Mendelian errors in nuclear families, Level 2 checks for all other Mendelian errors using the genotype elimination algorithm. CIDR runs Level 0 and 1 checks on a data set and removes Mendelian inconsistencies. After clearing up Level 0 and Level 1 errors, Level 2 checks are run to make sure that the data are free of Mendelian inconsistencies.

In our simulation study, we explore three questions:

1. How often are genotyping errors detected?
2. How often are these rules applied?
3. How often are these rules applied correctly?

We evaluate these questions via simulation study. In our simulation study, first we simulate error-free marker data for a single SNP (single nucleotide polymorphism) for 20,000 nuclear families (S1 File) with sibship sizes 2 to 6 [24] and SNP minor allele frequency (MAF) taking on the values 0.5, 0.4, 0.3, 0.2, 0.1 [25]. These simulations were done using the SIMULATE program [26]. Genotypes were simulated for all pedigree members. Secondly, we add in genotype errors using Mega2 [27]; this requires specification of the probability model for introducing errors, and the error rate. We introduce errors by picking a genotype at random with probability 0.01, and then changing the true genotype to one of the others with equal probability (for more see [28]. After introducing genotyping errors in data, we run Level 1 of PedCheck [20] to find the true underlying errors. Now we have two matched data sets: the original error free one, and the second one containing errors. After running PedCheck, we compute the percent of time genotype errors are detected in siblings, parents, or either. We also tabulate the percent of time each rule is applied. Finally, we compute how often each rule is applied correctly. We consider a rule to have been applied correctly if the genotype it zeroed out is a truly erroneous genotype.

## Results

**Fig 1:**
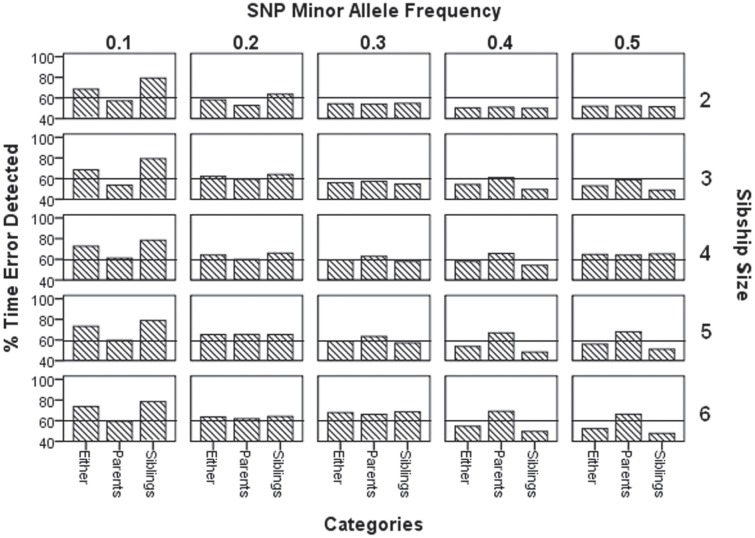
Percent of time a genotyping error is detected.

We present the results as Figures—for detailed counts, please see the supplemental tables. Fig 1 and S1 Table shows the percent of time genotyping errors are detected in siblings, parents, and in either. Using data set (S1 File), we observe that for sibship size 2 with MAF 0.3–0.5, error detection rates in parents and in siblings are very similar, while for sibship sizes 3 to 6, errors in parents are detected more often than errors in siblings. As the SNP becomes less polymorphic (i.e., MAF 0.1–0.2), errors in siblings are detected more often than errors in parents for all sibship sizes.

**Fig 2:**
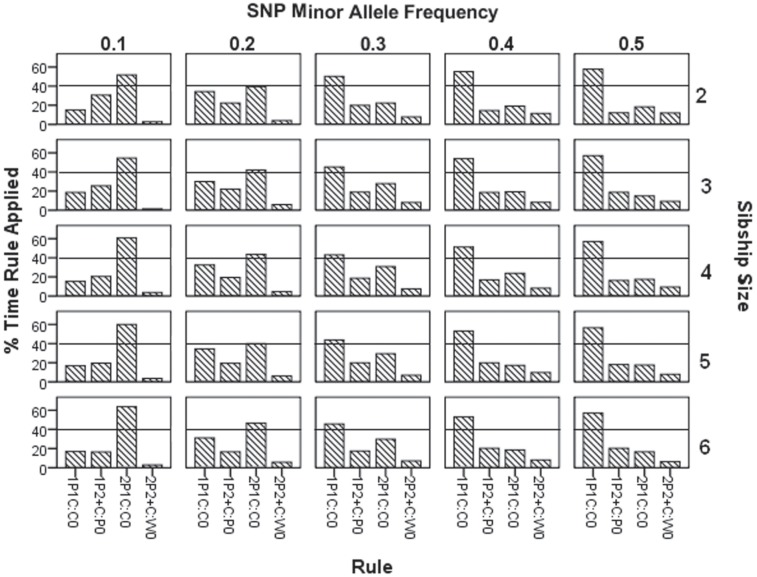
Percent of time each rule is applied.

For each genotype error that is detected, a CIDR rule, as defined in Table 1, is triggered (Fig 2 and S2 Table). Rules 1P1C:C_0_ and 2P1C:C_0_ are inversely proportional in application. As the MAF decreases from 0.5 to 0.1, the frequency of application of rule 1P1C:C_0_ decreases from ~58% to ~18%, while that of rule 2P1C:C_0_ increases from ~18% to ~60%. Rule 2P2+C:W_0_ is only applied 1–12% of the time. For MAF 0.1–0.2, rule 2P2+C:W_0_ is applied 1–6% of the time, and for MAF 0.3–0.5, rule 2P2+C:W_0_ is applied 6–12% of the time. Similarly, overall, rule 1P2+C:P_0_ is applied from 12% to 31% of the time. It is applied most frequently when the MAF is 0.1 and the sibship size is 2.

**Fig 3:**
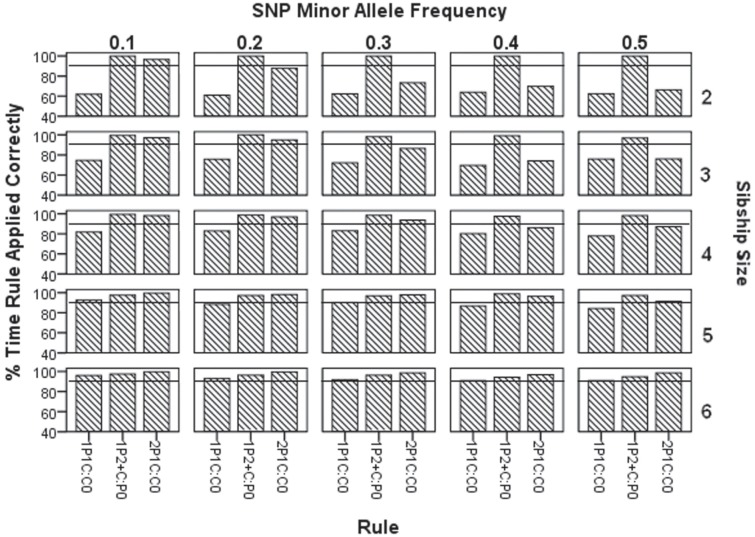
Percent of time each rule is applied correctly.

Fig 3 and S3 Table show how often each rule is applied correctly. Since rule 2P2+C:W_0_ zeros out the whole pedigree if it contains one or more genotyping error, it is always “correctly” applied. So we excluded rule 2P2+C:W_0_ from Fig 3 and S3 Table.

For rule 1P1C:C_0_, we observe in Fig 3 that for sibship size 2, the action taken is 61–64% correct. When we increase the sibship size from 2 to 3, then the percentage of the correctness of rule 1P1C:C_0_ also increases to 70–76%. Similarly in the same fashion, the correctness of rule 1P1C:C_0_ is 78–83%, 84–92% and 91–96% for sibship sizes 4, 5 and 6 respectively. In addition to noting this systematic increase in correctness of rule 1P1C:C_0_ with increase in sibship size, it is also important to note that there are not any drastic changes in the correctness of rule 1P1C:C_0_ with change in MAF within a given sibship size.

Rule 1P2+C:P_0_ is correctly applied 100% of the time for sibships of size 2, regardless of the MAF. As the sibship size increases, the frequency of correct application only declines very slightly: for sibship size 6, rule 1P2+C:P_0_ is applied correctly 94–97% of the time.

For rule 2P1C:C_0_, Fig 3 shows that for sibship size 2 with MAF 0.5, action taken for rule 2P1C:C_0_ (see Table 1) is 66% correct. For the same sibship size, the percentage of correctness of action taken for rule 2P1C:C_0_ is 70%, 73%, 88% and 97% respectively for MAF 0.4, 0.3, 0.2 and 0.1. In similar fashion when sibship size increases, the percentage of the correctness of action taken for rule 2P1C:C_0_ also increases, whereas the percentage of correctness of action are 66%, 76%, 87%, 91% and 99% respectively for sibship size 2, 3, 4, 5 and 6 with MAF 0.5. The action taken for rule 2P1C:C_0_ is highly correct for higher sibship sizes and also for low MAF when the sibships are smaller.

## Conclusion and discussion

In this study, we simulated data for nuclear families to examine the behavior of the CIDR data cleaning rules (Table 1). These rules are determined by the results of running PedCheck [19] to detect Mendelian inconsistencies. We examined how often a given error is detected, how often the rules are applied, and how often each rule is correctly applied.

Fig 1 shows how often errors are detected, and indicates that usually more errors are detected in parents than in siblings when MAF is 0.3–0.5. Similarly, when MAF is smaller (0.1–0.2), more errors are detected in siblings than parents. Overall true errors are detected at the rate of 51–74%. Douglas et al. [29] derived at the rate of 30–48% for 2 alleles model and 51–74% for 4 alleles model.

Fig 2 shows how often each rule is applied, and indicates that rule 1P1C:C_0_ is applied most frequently in nuclear families for MAF 0.3–0.5. Similarly, for SNP allele frequencies 0.1–0.2, rule 2P1C:C_0_ is applied most frequently, while rule 1P2+C:P_0_ is applied moderately (15–20%). Rule 2P2+C:W_0_ is always the least frequently applied rule (1–12%) across all MAF values and sibship sizes.

Fig 3 shows that how often actions taken by the CIDR rules shown in Table 1 are correct. Note that rule 2P2+C:W_0_ is excluded from Fig 3 because the concept of ‘correctness’ is not applicable to it if correctness means ‘the rule correctly zeroed out *only* the erroneous genotype’. Rule 2P2+C:W_0_ zeros out all the genotypes for the entire family. So while it does zero out the erroneous genotype, it also zeros out several correct genotypes.

Rule 1P2+C:P_0_ is almost always applied correctly (94–100%) as it is always correctly applied when there is one detectable true error in the nuclear family, and the underlying true error is in the parents (Fig 3). Alternatively, if there is only one true error in the pedigree in a single child, then it will not trigger rule 1P2+C:P_0_ because it will not cause one parent to be inconsistent with 2 or more children. When rule 1P2+C:P_0_ is applied less than 100% correctly, this is due to more than one true (and detectable) error occurring within a given family, which is a rare event in the smaller sibship sizes.

Rule 1P1C:C_0_ performs most incorrectly on smaller sibships (Fig 3) and is applied most frequently when it is extremely wrong (Fig 2), and is consistently more frequently wrong than the other rules. Rule 2P1C:C_0_ becomes more correctly applied as the MAF becomes smaller. CIDR’s rules are more often correctly applied as the sibship sizes get larger and MAF becomes smaller.

If we focus on where the rules are correctly applied (i.e. greater than 95% of the time), then we might come up with the following alternative rules, which should be better than CIDR’s rules:

1. Delete Rule 1P1C:C_0_, and instead zero out all the genotypes of the whole nuclear family when rule 1P1C:C_0_’s triggers apply.
2. Keep Rule 1P2+C:P_0_
3. Apply Rule 2P1C:C_0_ as a function of the MAF and sibship size; otherwise zero out all the genotypes of the whole family instead. Rule 2P1C:C_0_ is only to be applied to sibship of size 2 if the MAF ≤ 0.1; to size 3 if the MAF ≤ 0.2; to size 4 if the MAF ≤ 0.3; to size 5 if the MAF ≤ 0.4; and to size 6 for all values of the MAF.
4. Keep Rule 2P2+C:W_0_

We can also use another alternative approach which might be better than the CIDR’s rules—we may use Pedcheck’s Level 4 checking, and then zero out the genotype of any person whose alternative genotypes have at least one odds ratio of 1.0. Bedzioch et al. [30] examines genotyping errors using Level 4 of PEDCHECK for 4 data sets and conclude that Level 4 checking worked quite well (even when PEDCHECK did not indicate the most probable genotyping error in a few cases).

## Supporting information

S1 File Data set. (PDF)

S1 Table Percent of time genotype errors detected. The denominator is the number of actual errors present and the numerator is the number of errors detected by PedCheck.
(DOCX)

S2 Table Percent of time each rule is applied. (DOCX)

S3 Table Percent of time each rule is correctly applied. The denominator is the number of times each rule is applied and the numerator is the number of times each rule is applied correctly.
(DOCX)
