# Supplementary material for: Rules for resolving Mendelian inconsistencies in nuclear pedigrees typed for two-allele markers
Source: PLoS One. 2017 Mar 2;12(3):e0172807. doi: 10.1371/journal.pone.0172807 (PMC5333839; doi:10.1371/journal.pone.0172807)
Supplement: S1 File — (PDF) [file pone.0172807.s001.pdf]

3	1	0	1	1	3
17	3	0	0	3	2
31	1	1	0	0	3
44	1	0	1	1	3
71	1	0	1	1	3
103	1	1	0	0	3
123	1	1	0	0	3
126	1	0	1	1	3
150	1	0	1	1	3
155	1	0	0	1	1
170	3	0	3	0	2
197	1	0	1	1	3
227	1	0	0	1	1
241	1	0	1	1	3
285	1	0	0	1	1
326	3	0	0	3	2
328	1	0	1	1	3
335	1	1	0	0	3
342	1	1	0	0	3
343	1	1	0	0	3
353	3	0	0	3	2
370	1	0	0	1	1
397	1	0	1	0	1
432	1	1	0	0	3
491	3	0	0	3	2
514	3	0	0	3	2
531	1	0	0	1	1
563	1	1	0	0	3
574	3	0	0	3	2
578	3	0	3	0	2
607	1	1	0	0	3
661	2	0	0	2	2
677	1	0	1	1	3
701	1	1	0	0	3
733	1	1	0	0	3
767	1	0	1	1	3
783	1	0	0	1	1
786	1	0	1	1	3
827	1	1	0	0	3
900	1	0	1	0	1
916	1	1	0	0	3
921	1	0	0	1	1
925	3	0	0	3	2
934	3	0	3	0	2
936	1	0	1	0	1
950	1	1	0	0	3
951	1	1	0	0	3
964	2	1	1	1	3
967	1	1	0	0	3
1013	1	0	1	1	3

1041	1	0	1	1	3
1048	1	0	1	0	1
1058	1	1	0	0	3
1067	3	0	0	3	2
1069	3	0	3	0	2
1101	3	0	3	0	2
1141	1	1	0	0	3
1155	1	0	1	0	1
1169	1	0	1	1	3
1199	1	0	1	1	3
1204	1	1	0	0	3
1207	1	0	0	1	1
1266	3	0	3	0	2
1304	1	1	0	0	3
1347	1	0	1	0	1
1453	3	0	3	0	2
1488	1	0	0	1	1
1499	1	1	0	0	3
1518	1	1	0	0	3
1527	1	0	0	1	1
1545	3	0	0	3	2
1566	3	0	3	0	2
1578	1	1	0	0	3
1653	3	0	0	3	2
1699	1	1	0	0	3
1740	1	0	1	1	3
1750	1	0	1	1	3
1767	1	0	0	1	1
1769	1	0	1	1	3
1781	1	0	1	1	3
1824	1	0	1	0	1
1858	1	0	1	1	3
1869	3	0	0	3	2
1887	1	1	0	0	3
1985	1	0	1	1	3
1997	1	0	1	1	3
2016	3	0	3	0	2
2062	1	0	1	1	3
2117	3	0	3	0	2
2122	3	0	0	3	2
2221	1	0	1	1	3
2262	1	0	1	1	3
2293	2	0	0	2	2
2310	1	1	0	0	3
2339	3	0	0	3	2
2349	1	1	0	0	3
2361	1	0	1	0	1
2390	1	1	0	0	3
2433	1	0	1	0	1
2444	1	1	0	0	3

2459	1	1	0	0	3
2594	1	1	0	0	3
2612	1	0	1	0	1
2696	1	0	0	1	1
2713	1	0	1	0	1
2762	1	0	1	0	1
2778	2	2	0	0	4
2899	1	1	0	0	3
2920	1	1	0	0	3
2968	1	1	0	0	3
2985	1	0	1	1	3
2998	1	1	0	0	3
3016	1	0	1	0	1
3047	1	1	0	0	3
3051	1	1	0	0	3
3064	1	0	1	1	3
3097	1	1	0	0	3
3125	3	0	0	3	2
3138	1	1	0	0	3
3244	1	1	0	0	3
3265	3	0	0	3	2
3289	1	1	0	0	3
3348	2	0	0	2	2
3410	1	0	0	1	1
3416	1	1	0	0	3
3442	1	0	1	1	3
3447	1	0	1	1	3
3449	1	0	0	1	1
3470	1	0	0	1	1
3488	3	0	0	3	2
3491	2	0	2	0	2
3539	1	1	0	0	3
3548	1	0	1	0	1
3570	1	1	0	0	3
3691	1	1	0	0	3
3770	1	1	0	0	3
3785	3	0	3	0	2
3835	3	0	3	0	2
3853	1	0	1	0	1
3862	1	0	1	1	3
3886	1	0	0	1	1
3892	3	0	0	3	2
3904	3	0	0	3	2
3946	2	0	2	0	2
4060	1	1	0	0	3
4065	1	0	1	1	3
4105	1	1	0	0	3
4188	3	0	0	3	2
4213	1	0	1	0	1
4226	1	1	0	0	3

4262	1	1	0	0	3
4281	3	0	3	0	2
4311	1	1	0	0	3
4349	1	1	0	0	3
4368	1	1	0	0	3
4454	1	0	1	1	3
4497	1	0	1	0	1
4552	3	0	0	3	2
4558	1	0	0	1	1
4566	1	0	1	1	3
4584	2	0	0	2	2
4608	1	1	0	0	3
4625	2	0	0	2	2
4663	1	0	1	1	3
4704	3	0	3	0	2
4725	3	0	3	0	2
4773	1	1	0	0	3
4873	2	2	0	0	4
4924	1	1	0	0	3
4947	2	0	0	2	2
4968	1	1	0	0	3
4974	1	0	1	0	1
4988	1	1	0	0	3
4989	1	0	1	1	3
4998	3	0	3	0	2
5061	3	0	3	0	2
5063	3	0	3	0	2
5082	1	0	1	1	3
5085	1	1	0	0	3
5097	1	1	0	0	3
5117	1	0	1	0	1
5143	2	0	0	2	2
5156	1	0	0	1	1
5161	1	1	0	0	3
5182	1	1	0	0	3
5200	2	0	2	0	2
5249	3	0	0	3	2
5279	1	0	1	1	3
5300	1	0	1	1	3
5349	1	0	1	1	3
5443	1	0	1	1	3
5520	2	0	2	0	2
5529	1	1	0	0	3
5592	1	1	0	0	3
5601	1	0	1	1	3
5675	3	0	3	0	2
5702	1	1	0	0	3
5734	1	1	0	0	3
5785	1	0	1	0	1
5800	2	0	0	2	2

5848	1	0	1	1	3
5865	3	0	3	0	2
5888	1	0	0	1	1
5892	1	1	0	0	3
5935	1	0	1	1	3
5949	1	1	0	0	3
5985	1	0	1	1	3
6013	1	0	1	1	3
6036	1	0	0	1	1
6046	1	1	0	0	3
6063	1	0	1	1	3
6080	1	0	0	1	1
6100	1	0	0	1	1
6111	3	0	3	0	2
6128	1	0	1	0	1
6141	3	0	3	0	2
6204	1	0	1	1	3
6231	1	1	0	0	3
6336	1	1	0	0	3
6351	1	1	0	0	3
6374	1	0	0	1	1
6380	1	1	0	0	3
6386	1	0	1	1	3
6410	3	0	0	3	2
6447	1	0	1	1	3
6456	1	0	1	0	1
6514	3	0	3	0	2
6589	1	0	1	1	3
6639	1	0	1	1	3
6676	3	0	0	3	2
6679	2	0	0	2	2
6695	1	0	1	1	3
6724	1	0	1	1	3
6767	2	0	2	0	2
6773	1	1	0	0	3
6812	3	0	0	3	2
6842	1	0	1	1	3
6861	1	0	1	0	1
6865	1	1	0	0	3
6875	3	0	3	0	2
6885	1	0	0	1	1
6908	3	0	0	3	2
6926	1	0	1	1	3
6940	1	1	0	0	3
6956	1	1	0	0	3
6980	1	0	1	0	1
6986	1	0	0	1	1
6990	1	0	1	0	1
7017	3	0	3	0	2
7034	1	1	0	0	3

7040	1	0	1	1	3
7054	2	0	0	2	2
7060	3	0	3	0	2
7061	1	0	1	1	3
7088	1	0	1	1	3
7114	1	0	1	1	3
7215	1	0	1	1	3
7216	3	0	0	3	2
7242	1	0	1	1	3
7309	1	1	0	0	3
7332	1	1	0	0	3
7345	1	1	0	0	3
7357	1	0	0	1	1
7382	1	1	0	0	3
7416	1	1	0	0	3
7424	1	1	0	0	3
7432	1	0	1	1	3
7433	1	0	1	1	3
7438	2	2	0	0	4
7466	1	0	1	0	1
7540	1	1	0	0	3
7562	1	0	1	0	1
7592	1	0	1	1	3
7602	1	1	0	0	3
7606	1	0	1	1	3
7622	1	0	0	1	1
7665	1	0	1	1	3
7671	3	0	3	0	2
7699	1	1	0	0	3
7702	1	0	1	1	3
7738	1	1	0	0	3
7784	1	1	0	0	3
7812	1	0	1	1	3
7817	1	0	0	1	1
7822	3	0	3	0	2
7844	1	0	1	1	3
7864	1	0	1	1	3
7865	3	0	3	0	2
7877	1	0	1	1	3
7916	1	1	0	0	3
7921	2	0	0	2	2
7967	1	1	0	0	3
7980	3	0	3	0	2
7992	3	0	3	0	2
8034	3	0	0	3	2
8075	1	0	1	1	3
8108	1	0	1	1	3
8139	3	0	3	0	2
8141	1	1	0	0	3
8155	1	0	1	0	1

8172	1	0	1	1	3
8207	1	0	1	1	3
8264	3	0	3	0	2
8327	1	1	0	0	3
8372	1	0	1	1	3
8383	1	0	1	1	3
8422	1	0	1	1	3
8456	1	0	1	0	1
8459	2	2	0	0	4
8507	1	1	0	0	3
8523	1	1	0	0	3
8530	1	0	1	1	3
8552	1	1	0	0	3
8556	1	0	1	0	1
8577	3	0	3	0	2
8652	1	1	0	0	3
8662	1	0	1	1	3
8715	1	0	1	1	3
8737	1	0	1	1	3
8789	1	1	0	0	3
8848	1	0	0	1	1
8911	1	1	0	0	3
8922	1	1	0	0	3
8929	1	1	0	0	3
8983	1	1	0	0	3
9012	1	0	1	1	3
9039	1	0	0	1	1
9072	1	0	1	1	3
9084	1	0	1	1	3
9123	1	0	1	1	3
9127	3	0	3	0	2
9138	1	1	0	0	3
9149	1	0	1	1	3
9179	1	1	0	0	3
9180	1	0	1	1	3
9261	1	0	0	1	1
9272	3	0	3	0	2
9282	1	0	1	1	3
9313	3	0	3	0	2
9348	1	0	1	1	3
9380	1	0	1	0	1
9431	3	0	3	0	2
9500	1	0	1	1	3
9525	3	0	0	3	2
9557	1	1	0	0	3
9628	1	1	0	0	3
9682	3	0	3	0	2
9688	1	0	0	1	1
9696	1	0	1	0	1
9727	1	1	0	0	3

9731	1	0	0	1	1
9769	3	0	0	3	2
9874	2	0	2	0	2
9884	1	1	0	0	3
9966	2	0	2	0	2
9997	3	0	3	0	2
10021	1	0	1	1	3
10031	1	1	0	0	3
10079	1	1	0	0	3
10086	1	0	1	1	3
10104	1	0	1	0	1
10142	1	1	0	0	3
10147	3	0	0	3	2
10168	1	0	1	1	3
10249	1	0	1	1	3
10297	3	0	3	0	2
10340	1	1	0	0	3
10392	1	0	1	1	3
10394	2	0	2	0	2
10398	1	0	1	1	3
10399	1	0	1	1	3
10483	1	0	1	0	1
10484	1	0	0	1	1
10491	3	0	0	3	2
10525	1	1	0	0	3
10579	1	1	0	0	3
10613	1	0	1	0	1
10628	2	0	0	2	2
10654	3	0	3	0	2
10708	1	0	0	1	1
10718	1	0	1	1	3
10731	1	0	1	1	3
10733	1	1	0	0	3
10736	1	1	0	0	3
10751	1	0	1	1	3
10774	1	0	1	1	3
10777	1	0	1	1	3
10781	1	0	1	1	3
10783	1	0	1	1	3
10818	1	0	0	1	1
10840	1	0	1	1	3
10892	1	1	0	0	3
10900	1	0	1	1	3
10919	1	1	0	0	3
11061	1	0	1	1	3
11080	1	1	0	0	3
11081	1	0	1	1	3
11083	3	0	3	0	2
11128	1	1	0	0	3
11129	1	0	1	0	1

11158	1	1	0	0	3
11173	1	0	1	1	3
11258	1	0	1	0	1
11283	3	0	0	3	2
11352	3	0	0	3	2
11368	1	0	1	0	1
11370	1	1	0	0	3
11388	1	1	0	0	3
11413	1	0	1	0	1
11478	1	0	1	1	3
11494	1	0	0	1	1
11559	1	0	1	1	3
11595	1	0	1	0	1
11643	3	0	0	3	2
11651	1	0	1	1	3
11661	1	1	0	0	3
11698	3	0	3	0	2
11759	3	0	0	3	2
11761	1	0	1	1	3
11820	1	1	0	0	3
11828	1	1	0	0	3
11843	3	0	3	0	2
11890	1	0	0	1	1
11896	3	0	3	0	2
11899	1	0	1	1	3
11904	3	0	3	0	2
11956	1	1	0	0	3
12033	3	0	0	3	2
12059	1	0	0	1	1
12131	1	1	0	0	3
12150	1	1	0	0	3
12152	1	0	0	1	1
12197	1	1	0	0	3
12210	1	0	1	1	3
12263	3	0	0	3	2
12265	3	0	3	0	2
12269	3	0	0	3	2
12278	1	1	0	0	3
12282	1	1	0	0	3
12321	3	0	3	0	2
12342	1	0	1	1	3
12392	1	1	0	0	3
12413	1	1	0	0	3
12479	3	0	0	3	2
12534	1	0	1	0	1
12536	1	0	0	1	1
12618	1	1	0	0	3
12656	3	0	3	0	2
12658	1	1	0	0	3
12673	1	0	1	1	3

12712	1	1	0	0	3
12737	3	0	3	0	2
12745	2	0	0	2	2
12801	1	0	0	1	1
12814	1	1	0	0	3
12816	3	0	3	0	2
12828	1	0	1	1	3
12850	1	1	0	0	3
12867	1	1	0	0	3
12886	3	0	3	0	2
12896	3	0	0	3	2
12903	1	1	0	0	3
12913	1	1	0	0	3
12917	1	0	1	1	3
12921	1	0	1	1	3
12922	3	0	3	0	2
12925	1	0	0	1	1
12931	1	0	1	1	3
12943	1	0	1	0	1
12971	1	0	1	1	3
12987	1	0	0	1	1
13053	3	0	3	0	2
13143	1	1	0	0	3
13226	1	0	1	0	1
13260	1	1	0	0	3
13273	1	0	1	1	3
13390	1	1	0	0	3
13394	1	0	1	1	3
13430	3	0	3	0	2
13467	1	0	0	1	1
13485	1	1	0	0	3
13511	1	0	1	1	3
13512	1	1	0	0	3
13513	1	1	0	0	3
13515	1	0	1	1	3
13535	1	0	1	1	3
13557	3	0	0	3	2
13589	2	0	0	2	2
13600	3	0	0	3	2
13616	3	0	3	0	2
13649	1	1	0	0	3
13656	1	1	0	0	3
13713	1	0	1	1	3
13739	1	1	0	0	3
13749	1	0	0	1	1
13784	1	1	0	0	3
13791	1	0	1	0	1
13799	1	0	1	1	3
13815	1	0	1	1	3
13882	2	0	2	0	2

13911	1	1	0	0	3
13922	1	0	1	0	1
13946	1	0	1	1	3
13963	1	0	1	1	3
13971	1	1	0	0	3
13980	3	0	0	3	2
14009	1	0	1	1	3
14035	1	0	1	1	3
14072	3	3	0	0	4
14084	1	0	1	1	3
14101	1	0	1	1	3
14204	3	0	3	0	2
14223	1	1	0	0	3
14238	1	0	1	1	3
14295	1	1	0	0	3
14327	1	0	1	1	3
14331	1	0	1	1	3
14333	3	0	3	0	2
14341	1	0	1	0	1
14382	1	0	0	1	1
14391	1	0	0	1	1
14399	3	0	0	3	2
14405	1	1	0	0	3
14432	1	0	0	1	1
14443	3	0	0	3	2
14445	3	0	0	3	2
14543	1	0	1	1	3
14552	1	0	0	1	1
14592	1	1	0	0	3
14613	1	0	0	1	1
14643	1	0	1	1	3
14723	3	0	3	0	2
14764	3	0	3	0	2
14774	3	0	3	0	2
14778	1	0	1	1	3
14784	1	0	1	0	1
14859	3	0	0	3	2
14863	1	0	0	1	1
14869	1	1	0	0	3
14902	1	0	1	0	1
14915	1	0	0	1	1
14917	2	0	2	0	2
14942	3	0	3	0	2
14958	1	1	0	0	3
14994	1	0	0	1	1
15014	1	0	1	1	3
15020	1	0	1	1	3
15046	1	1	0	0	3
15065	1	0	1	0	1
15125	1	0	0	1	1

15128	1	0	0	1	1
15141	3	0	0	3	2
15162	1	1	0	0	3
15271	1	0	1	1	3
15312	1	0	1	1	3
15359	1	0	1	1	3
15483	3	0	0	3	2
15486	1	1	0	0	3
15501	3	0	3	0	2
15520	2	0	2	0	2
15528	1	0	1	1	3
15591	1	0	1	1	3
15638	3	0	3	0	2
15645	1	1	0	0	3
15652	1	0	1	0	1
15670	1	1	0	0	3
15693	2	2	0	0	4
15748	5	1	2	2	3
15756	3	0	3	0	2
15774	1	1	0	0	3
15788	1	1	0	0	3
15800	1	1	0	0	3
15855	1	0	1	1	3
15913	2	2	0	0	4
15922	3	0	0	3	2
15924	1	0	1	1	3
16107	1	0	1	0	1
16108	1	0	0	1	1
16118	3	0	3	0	2
16232	3	0	3	0	2
16255	1	1	0	0	3
16330	1	1	0	0	3
16361	3	0	0	3	2
16392	1	0	1	0	1
16439	1	1	0	0	3
16480	3	0	3	0	2
16492	1	0	1	1	3
16495	1	1	0	0	3
16717	1	1	0	0	3
16838	1	0	1	1	3
16859	1	0	0	1	1
16907	1	1	0	0	3
16908	3	0	0	3	2
16936	1	0	0	1	1
17021	1	0	1	1	3
17039	3	0	3	0	2
17061	3	0	0	3	2
17073	1	0	0	1	1
17091	3	0	0	3	2
17096	1	1	0	0	3

17183	3	0	3	0	2
17190	1	1	0	0	3
17191	1	1	0	0	3
17195	3	0	0	3	2
17196	1	0	1	1	3
17231	1	1	0	0	3
17263	1	1	0	0	3
17291	1	1	0	0	3
17331	1	0	1	0	1
17335	3	0	3	0	2
17363	1	0	1	1	3
17380	3	0	3	0	2
17386	1	0	1	0	1
17387	1	1	0	0	3
17392	1	1	0	0	3
17405	3	0	3	0	2
17407	2	2	0	0	4
17453	3	0	3	0	2
17458	1	0	1	1	3
17478	3	3	0	0	4
17490	1	0	1	1	3
17530	1	1	0	0	3
17628	1	0	1	0	1
17651	3	0	1	2	2
17878	1	1	0	0	3
17884	1	0	0	1	1
17909	1	1	0	0	3
17986	1	1	0	0	3
18011	1	0	1	1	3
18076	1	0	1	0	1
18093	1	1	0	0	3
18129	2	0	2	0	2
18159	1	1	0	0	3
18186	3	0	0	3	2
18193	3	0	0	3	2
18228	1	0	1	1	3
18240	3	0	3	0	2
18282	3	0	3	0	2
18318	3	0	1	2	2
18342	3	0	0	3	2
18363	1	0	1	1	3
18369	1	0	1	1	3
18396	3	0	3	0	2
18405	2	1	1	1	3
18464	1	0	0	1	1
18467	1	0	1	1	3
18501	1	1	0	0	3
18561	1	0	1	1	3
18563	1	0	1	1	3
18588	3	0	3	0	2

18593	1	1	0	0	3
18603	1	0	0	1	1
18605	1	0	0	1	1
18631	1	0	1	1	3
18644	3	0	0	3	2
18649	1	0	1	1	3
18685	1	0	0	1	1
18747	1	0	0	1	1
18820	1	0	1	1	3
18822	1	0	1	0	1
18888	1	0	1	0	1
18917	2	0	0	2	2
18923	1	1	0	0	3
18937	3	0	0	3	2
18952	2	2	0	0	4
18969	3	0	3	0	2
19065	1	0	1	1	3
19068	1	1	0	0	3
19088	3	0	0	3	2
19136	2	0	2	0	2
19153	1	1	0	0	3
19156	1	1	0	0	3
19178	3	0	3	0	2
19212	3	0	3	0	2
19242	3	0	3	0	2
19265	1	0	1	1	3
19330	2	0	0	2	2
19453	1	1	0	0	3
19515	1	1	0	0	3
19535	1	0	1	1	3
19576	1	0	0	1	1
19609	1	1	0	0	3
19616	1	0	1	1	3
19627	2	0	2	0	2
19647	1	0	1	1	3
19653	1	0	0	1	1
19660	1	1	0	0	3
19681	1	0	1	1	3
19704	1	1	0	0	3
19719	1	0	1	0	1
19766	1	0	1	1	3
19790	3	0	3	0	2
19796	3	0	0	3	2
19799	1	0	1	0	1
19816	1	0	1	1	3
19831	1	1	0	0	3
19834	1	1	0	0	3
19887	1	0	1	0	1
19898	1	0	0	1	1
19925	3	0	0	3	2

19981	1	0	1	1	3
20000	1	1	0	0	3
