# Supplementary material for: Rules for resolving Mendelian inconsistencies in nuclear pedigrees typed for two-allele markers
Source: PLoS One. 2017 Mar 2;12(3):e0172807. doi: 10.1371/journal.pone.0172807 (PMC5333839; doi:10.1371/journal.pone.0172807)
Supplement: S1 Table — The denominator is the number of actual errors present and the numerator is the number of errors detected by PedCheck. (DOCX) [file pone.0172807.s002.docx]

S1 Table: Percent of time genotype errors detected. The denominator is the number of actual errors present and the numerator is the number of errors detected by PedCheck.

| Sibship Size | Categories | SNP Allele Frequency | | | | |
| --- | --- | --- | --- | --- | --- | --- |
|  |  | 0.1 | 0.2 | 0.3 | 0.4 | 0.5 |
| 2 | Siblings | $\frac{333}{420}\left(79\%\right)$ | $\frac{266}{418}\left(64\%\right)$ | $\frac{216}{394}\left(55\%\right)$ | $\frac{194}{388}\left(50\%\right)$ | $\frac{195}{378}\left(52\%\right)$ |
|  | Parents | $\frac{228}{398}\left(57\%\right)$ | $\frac{213}{404}\left(53\%\right)$ | $\frac{239}{444}\left(54\%\right)$ | $\frac{203}{398}\left(51\%\right)$ | $\frac{210}{401}\left(52\%\right)$ |
|  | Either | $\frac{561}{818}\left(69\%\right)$ | $\frac{479}{822}\left(58\%\right)$ | $\frac{455}{838}\left(54\%\right)$ | $\frac{397}{786}\left(51\%\right)$ | $\frac{405}{779}\left(52\%\right)$ |
| 3 | Siblings | $\frac{471}{594}\left(79\%\right)$ | $\frac{361}{564}\left(64\%\right)$ | $\frac{318}{581}\left(55\%\right)$ | $\frac{302}{609}\left(50\%\right)$ | $\frac{277}{566}\left(49\%\right)$ |
|  | Parents | $\frac{231}{429}\left(54\%\right)$ | $\frac{216}{364}\left(59\%\right)$ | $\frac{239}{415}\left(58\%\right)$ | $\frac{278}{456}\left(61\%\right)$ | $\frac{224}{380}\left(59\%\right)$ |
|  | Either | $\frac{702}{1023}\left(69\%\right)$ | $\frac{577}{928}\left(62\%\right)$ | $\frac{557}{996}\left(56\%\right)$ | $\frac{580}{1065}\left(54\%\right)$ | $\frac{501}{946}\left(53\%\right)$ |
| 4 | Siblings | $\frac{653}{832}\left(78\%\right)$ | $\frac{513}{777}\left(66\%\right)$ | $\frac{465}{804}\left(58\%\right)$ | $\frac{435}{804}\left(54\%\right)$ | $\frac{407}{624}\left(65\%\right)$ |
|  | Parents | $\frac{250}{409}\left(61\%\right)$ | $\frac{225}{375}\left(60\%\right)$ | $\frac{249}{396}\left(63\%\right)$ | $\frac{264}{402}\left(66\%\right)$ | $\frac{267}{416}\left(64\%\right)$ |
|  | Either | $\frac{903}{1241}\left(73\%\right)$ | $\frac{738}{1152}\left(64\%\right)$ | $\frac{714}{1200}\left(60\%\right)$ | $\frac{699}{1206}\left(58\%\right)$ | $\frac{674}{1040}\left(65\%\right)$ |
| 5 | Siblings | $\frac{784}{994}\left(79\%\right)$ | $\frac{634}{971}\left(65\%\right)$ | $\frac{548}{959}\left(57\%\right)$ | $\frac{496}{1026}\left(48\%\right)$ | $\frac{513}{1002}\left(51\%\right)$ |
|  | Parents | $\frac{244}{409}\left(60\%\right)$ | $\frac{265}{405}\left(65\%\right)$ | $\frac{245}{386}\left(63\%\right)$ | $\frac{281}{420}\left(67\%\right)$ | $\frac{281}{414}\left(68\%\right)$ |
|  | Either | $\frac{1028}{1403}\left(73\%\right)$ | $\frac{899}{1376}\left(65\%\right)$ | $\frac{793}{1345}\left(59\%\right)$ | $\frac{777}{1446}\left(54\%\right)$ | $\frac{794}{1416}\left(56\%\right)$ |
| 6 | Siblings | $\frac{946}{1206}\left(78\%\right)$ | $\frac{751}{1171}\left(64\%\right)$ | $\frac{682}{996}\left(68\%\right)$ | $\frac{578}{1164}\left(50\%\right)$ | $\frac{589}{1231}\left(48\%\right)$ |
|  | Parents | $\frac{229}{385}\left(59\%\right)$ | $\frac{228}{368}\left(62\%\right)$ | $\frac{262}{396}\left(66\%\right)$ | $\frac{275}{398}\left(69\%\right)$ | $\frac{261}{394}\left(66\%\right)$ |
|  | Either | $\frac{1175}{1591}\left(74\%\right)$ | $\frac{979}{1539}\left(64\%\right)$ | $\frac{944}{1392}\left(68\%\right)$ | $\frac{853}{1562}\left(55\%\right)$ | $\frac{850}{1625}\left(52\%\right)$ |
