# Supplementary material for: Rules for resolving Mendelian inconsistencies in nuclear pedigrees typed for two-allele markers
Source: PLoS One. 2017 Mar 2;12(3):e0172807. doi: 10.1371/journal.pone.0172807 (PMC5333839; doi:10.1371/journal.pone.0172807)
Supplement: S2 Table — (DOCX) [file pone.0172807.s003.docx]

**S2 Table**: Percent of time each rule is applied.

| Sibship Size | Rule | SNP Allele Frequency | | | | |
| --- | --- | --- | --- | --- | --- | --- |
|  |  | 0.1 | 0.2 | 0.3 | 0.4 | 0.5 |
| 2 | 1P1C:C_0_ | $\frac{84}{561}\left(15\%\right)$ | $\frac{164}{479}\left(34\%\right)$ | $\frac{228}{455}\left(50\%\right)$ | $\frac{219}{397}\left(55\%\right)$ | $\frac{234}{405}\left(58\%\right)$ |
|  | 1P2+C:P_0_ | $\frac{172}{561}\left(31\%\right)$ | $\frac{107}{479}\left(22\%\right)$ | $\frac{91}{455}\left(20\%\right)$ | $\frac{57}{397}\left(14\%\right)$ | $\frac{49}{405}\left(12\%\right)$ |
|  | 2P1C:C_0_3 | $\frac{290}{561}\left(52\%\right)$ | $\frac{189}{479}\left(39\%\right)$ | $\frac{101}{455}\left(22\%\right)$ | $\frac{76}{397}\left(19\%\right)$ | $\frac{74}{405}\left(18\%\right)$ |
|  | 2P2+C:W_0_ | $\frac{15}{561}\left(3\%\right)$ | $\frac{19}{479}\left(4\%\right)$ | $\frac{35}{455}\left(8\%\right)$ | $\frac{45}{397}\left(11\%\right)$ | $\frac{48}{405}\left(12\%\right)$ |
| 3 | 1P1C:C_0_ | $\frac{130}{702}\left(19\%\right)$ | $\frac{173}{577}\left(30\%\right)$ | $\frac{252}{557}\left(45\%\right)$ | $\frac{312}{580}\left(54\%\right)$ | $\frac{286}{501}\left(57\%\right)$ |
|  | 1P2+C:P_0_ | $\frac{179}{702}\left(25\%\right)$ | $\frac{128}{577}\left(22\%\right)$ | $\frac{105}{557}\left(19\%\right)$ | $\frac{108}{580}\left(19\%\right)$ | $\frac{94}{501}\left(19\%\right)$ |
|  | 2P1C:C_0_ | $\frac{383}{702}\left(55\%\right)$ | $\frac{242}{577}\left(42\%\right)$ | $\frac{155}{557}\left(28\%\right)$ | $\frac{112}{580}\left(19\%\right)$ | $\frac{75}{501}\left(15\%\right)$ |
|  | 2P2+C:W_0_ | $\frac{10}{702}\left(1\%\right)$ | $\frac{34}{577}\left(6\%\right)$ | $\frac{45}{557}\left(8\%\right)$ | $\frac{48}{580}\left(8\%\right)$ | $\frac{46}{501}\left(9\%\right)$ |
| 4 | 1P1C:C_0_ | $\frac{137}{903}\left(15\%\right)$ | $\frac{239}{738}\left(32\%\right)$ | $\frac{307}{714}\left(43\%\right)$ | $\frac{358}{699}\left(51\%\right)$ | $\frac{384}{674}\left(57\%\right)$ |
|  | 1P2+C:P_0_ | $\frac{184}{903}\left(20\%\right)$ | $\frac{143}{738}\left(19\%\right)$ | $\frac{133}{714}\left(19\%\right)$ | $\frac{118}{699}\left(17\%\right)$ | $\frac{110}{674}\left(16\%\right)$ |
|  | 2P1C:C_0_ | $\frac{548}{903}\left(61\%\right)$ | $\frac{322}{738}\left(44\%\right)$ | $\frac{220}{714}\left(31\%\right)$ | $\frac{166}{699}\left(24\%\right)$ | $\frac{117}{674}\left(17\%\right)$ |
|  | 2P2+C:W_0_ | $\frac{34}{903}\left(4\%\right)$ | $\frac{34}{738}\left(5\%\right)$ | $\frac{54}{714}\left(8\%\right)$ | $\frac{57}{699}\left(8\%\right)$ | $\frac{63}{674}\left(9\%\right)$ |
| 5 | 1P1C:C_0_ | $\frac{173}{1028}\left(17\%\right)$ | $\frac{310}{899}\left(34\%\right)$ | $\frac{347}{793}\left(44\%\right)$ | $\frac{413}{777}\left(53\%\right)$ | $\frac{449}{794}\left(57\%\right)$ |
|  | 1P2+C:P_0_ | $\frac{202}{1028}\left(20\%\right)$ | $\frac{174}{899}\left(19\%\right)$ | $\frac{158}{793}\left(20\%\right)$ | $\frac{155}{777}\left(20\%\right)$ | $\frac{144}{794}\left(18\%\right)$ |
|  | 2P1C:C_0_ | $\frac{617}{1028}\left(60\%\right)$ | $\frac{360}{899}\left(40\%\right)$ | $\frac{233}{793}\left(29\%\right)$ | $\frac{134}{777}\left(17\%\right)$ | $\frac{139}{794}\left(18\%\right)$ |
|  | 2P2+C:W_0_ | $\frac{36}{1028}\left(4\%\right)$ | $\frac{55}{899}\left(6\%\right)$ | $\frac{55}{793}\left(7\%\right)$ | $\frac{75}{777}\left(10\%\right)$ | $\frac{62}{794}\left(8\%\right)$ |
| 6 | 1P1C:C_0_ | $\frac{199}{1175}\left(17\%\right)$ | $\frac{305}{979}\left(31\%\right)$ | $\frac{431}{944}\left(46\%\right)$ | $\frac{452}{853}\left(53\%\right)$ | $\frac{484}{850}\left(57\%\right)$ |
|  | 1P2+C:P_0_ | $\frac{193}{1175}\left(16\%\right)$ | $\frac{162}{979}\left(17\%\right)$ | $\frac{165}{944}\left(17\%\right)$ | $\frac{173}{853}\left(20\%\right)$ | $\frac{172}{850}\left(20\%\right)$ |
|  | 2P1C:C_0_ | $\frac{749}{1175}\left(64\%\right)$ | $\frac{456}{979}\left(47\%\right)$ | $\frac{281}{944}\left(30\%\right)$ | $\frac{159}{853}\left(19\%\right)$ | $\frac{141}{850}\left(17\%\right)$ |
|  | 2P2+C:W_0_ | $\frac{34}{1175}\left(3\%\right)$ | $\frac{56}{979}\left(6\%\right)$ | $\frac{67}{944}\left(7\%\right)$ | $\frac{69}{853}\left(8\%\right)$ | $\frac{53}{850}\left(6\%\right)$ |
