# Supplementary material for: Rules for resolving Mendelian inconsistencies in nuclear pedigrees typed for two-allele markers
Source: PLoS One. 2017 Mar 2;12(3):e0172807. doi: 10.1371/journal.pone.0172807 (PMC5333839; doi:10.1371/journal.pone.0172807)
Supplement: S3 Table — The denominator is the number of times each rule is applied and the numerator is the number of times each rule is applied correctly. (DOCX) [file pone.0172807.s004.docx]

S3 Table: Percent of time each rule is correctly applied. The denominator is the number of times each rule is applied and the numerator is the number of times each rule is applied correctly.

| Sibship Size | Rule # | SNP Allele Frequency | | | | |
| --- | --- | --- | --- | --- | --- | --- |
|  |  | 0.1 | 0.2 | 0.3 | 0.4 | 0.5 |
| 2 | 1P1C:C_0_ |  |  |  |  |  |
|  | 1P2+C:P_0_ |  |  |  |  |  |
|  | 2P1C:C_0_ |  |  |  |  |  |
| 3 | 1P1C:C_0_ |  |  |  |  |  |
|  | 1P2+C:P_0_ |  |  |  |  |  |
|  | 2P1C:C_0_ |  |  |  |  |  |
| 4 | 1P1C:C_0_ |  |  |  |  |  |
|  | 1P2+C:P_0_ |  |  |  |  |  |
|  | 2P1C:C_0_ |  |  |  |  |  |
| 5 | 1P1C:C_0_ |  |  |  |  |  |
|  | 1P2+C:P_0_ |  |  |  |  |  |
|  | 2P1C:C_0_ |  |  |  |  |  |
| 6 | 1P1C:C_0_ |  |  |  |  |  |
|  | 1P2+C:P_0_ |  |  |  |  |  |
|  | 2P1C:C_0_ |  |  |  |  |  |
